# Mobile Apps for Wound Assessment and Monitoring: Limitations, Advancements and Opportunities

**DOI:** 10.1007/s10916-024-02091-x

**Published:** 2024-08-24

**Authors:** Muhammad Ashad Kabir, Sabiha Samad, Fahmida Ahmed, Samsun Naher, Jill Featherston, Craig Laird, Sayed Ahmed

**Affiliations:** 1 School of Computing, Mathematics and Engineering, Charles Sturt University, Bathurst, 2795 NSW Australia; 2 Department of Computer Science and Engineering, Chittagong University of Engineering and Technology, Chattogram, 4349 Chattogram Bangladesh; 3 School of Medicine, Cardiff University, Cardiff, CF14 4YS Wales United Kingdom; 4 Principal Pedorthist, Walk Easy Pedorthics Pty. Ltd., Tamworth, 2340 NSW Australia; 5 Principal Pedorthist, Foot Balance Technology Pty Ltd, Westmead, 2145 NSW Australia; 6 Offloading Clinic, Nepean Hospital, Kingswood, 2750 NSW Australia

**Keywords:** Wound, Ulcer, Mobile, Smartphone, Apps, Artificial intelligence

## Abstract

With the proliferation of wound assessment apps across various app stores and the increasing integration of artificial intelligence (AI) in healthcare apps, there is a growing need for a comprehensive evaluation system. Current apps lack sufficient evidence-based reliability, prompting the necessity for a systematic assessment. The objectives of this study are to evaluate the wound assessment and monitoring apps, identify limitations, and outline opportunities for future app development. An electronic search across two major app stores (Google Play store, and Apple App Store) was conducted and the selected apps were rated by three independent raters. A total of 170 apps were discovered, and 10 were selected for review based on a set of inclusion and exclusion criteria. By modifying existing scales, an app rating scale for wound assessment apps is created and used to evaluate the selected ten apps. Our rating scale evaluates apps’ functionality and software quality characteristics. Most apps in the app stores, according to our evaluation, do not meet the overall requirements for wound monitoring and assessment. All the apps that we reviewed are focused on practitioners and doctors. According to our evaluation, the app **ImitoWound** got the highest mean score of 4.24. But this app has 7 criteria among our 11 functionalities criteria. Finally, we have recommended future opportunities to leverage advanced techniques, particularly those involving artificial intelligence, to enhance the functionality and efficacy of wound assessment apps. This research serves as a valuable resource for future developers and researchers seeking to enhance the design of wound assessment-based applications, encompassing improvements in both software quality and functionality.

## Introduction

A wound is characterized as a disruption or injury to the skin, resulting in a break in the skin’s integrity, leading to a complex and dynamic process of healing [1]. An ulcer, a specific type of wound, manifests as an open, painful sore that can range from minor skin injuries to severe scars penetrating muscle tissue, and, in severe cases, exposing bones and joints, potentially leading to chronic wounds and significant morbidity [2]. Various types of ulcers, such as pressure ulcers (also known as pressure injuries or bedsores) and diabetic foot ulcers (DFUs), pose substantial challenges in healthcare settings due to their high prevalence, complexity, and potential for severe complications [3, 4]. Pressure ulcers, for example, are a common problem in immobile patients, while DFUs are a major concern for individuals with diabetes, highlighting the need for effective prevention and management strategies [5, 6].

Pressure ulcers, prevalent in intensive care units (ICUs) in Brazilian hospitals, afflict 17.2% to 41% of patients [7, 8]. In the United States, ICUs report prevalence rates ranging from 8.8% to 12.1%, while acute care units experience rates as high as 22% [9]. DFUs are equally concerning, affecting approximately 50,000 individuals daily in Australia alone and resulting in significant healthcare utilization, including hospitalizations, amputations, and costs amounting to approximately $1 billion annually [10–12]. The long-term consequences of DFUs are alarming, with over 20 million individuals globally having undergone lower limb amputations [13], a number projected to double by 2030 [14]. Moreover, DFUs not only impact physical health but also have profound implications for mental well-being, affecting patients, families, workplaces, and communities [10]. Studies have shown that more than 70% of individuals with DFUs experience disease progression over a five-year period, highlighting the chronic and debilitating nature of this condition [15]. Additionally, untreated skin ulcers are susceptible to infection, leading to prolonged healing times and potentially exacerbating the underlying condition.

This necessitates a physician’s and health team’s systematic monitoring of the wound’s progress. Wound assessment comprises both qualitative (appearance of the wound, boundaries, perilesional skin) and quantitative evaluations (length, width, area, depth, etc.) [16]. In the latter case, an effective method or technique should allow repeatable assessments from one inspector to the next, as well as responsiveness to minor wound changes. However, in-person evaluation and follow-up by trained professionals are not always possible, particularly when the patient does not have access to special transportation to a specialized care center or has no family member or resident in a remote place. Wound evaluation can now be optimized by allowing an interprofessional team to remotely see, analyze, and monitor wound evolution through apps, thanks to the rise of mobile health (mHealth) and the prevalence of mobile devices in clinics and hospitals [17].

In contemporary clinical settings, the widespread integration of smartphones and tablets is increasingly prevalent [16]. The utilization of mobile applications (apps) in wound care holds considerable promise, offering the ability to capture images of wounds using smartphone cameras to calculate the size and surface area. Such apps have the potential to significantly enhance nursing practices and improve patient care [18]. A recent study in a health service in New South Wales, Australia, reported improvements in wound documentation with the use of an AI-enabled mobile app [19]. AI-powered mobile imaging enables practitioners to detect and classify the stages of pressure injury wounds [20] and to measure size [21], while also remotely analyzing and tracking the progression of wounds over time [22–24]. This technology enhances the accuracy and efficiency of wound management [25]. Implementing standardised wound assessment and documentation processes through mobile apps can significantly enhance the quality of wound care management. These mobile apps enable care providers to access comprehensive patient information in a centralized location, available anytime and anywhere. Additionally, decision-makers benefit from access to statistics, analytics, and resource utilization data, facilitating informed decision-making and improving overall healthcare outcomes [26].

Over the past decade, the proliferation of mobile health (mHealth) apps, particularly those focused on wound quality assessment, has been notable. Many apps in this domain have exhibited various challenges pertaining to usability, design, and functionality [27–29]. Content et al. [30] emphasised the need for rigorous validation processes in wound care mobile applications. Ensuring the validity and reliability of these apps is crucial. Inadequately validated apps may expose users to biased or inaccurate information.

The stimulus for this study was twofold. Firstly, the escalating global burden of chronic wounds, coupled with the rapid integration of mobile devices in clinical settings, sparked our interest in evaluating the efficacy and usability of mHealth apps in wound care. Secondly, the proliferation of mobile health technologies over the past decade has seen a surge in apps designed for wound assessment, yet challenges such as usability, design, and functionality persist. Through our review, we aimed to provide a comprehensive assessment of existing apps, highlighting their strengths, weaknesses, and potential impact on clinical practice. Our objective is to contribute valuable insights that inform healthcare professionals, researchers, and developers in refining these technologies to better serve patient needs and improve overall healthcare outcomes.

Koepp et al. [17] conducted a qualitative synthesis of research studies focusing on the development of wound apps. In [31], the focus was on patient-centric wound care mobile apps, with only three apps identified and their quality assessed using the Mobile App Rating Scale (MARS) [32]. In contrast, our study did not confine its scope solely to patient-centric apps; instead, we identified wound apps primarily tailored for healthcare practitioners. Moreover, our evaluation of these apps was conducted utilizing a custom app rating scale. Unlike MARS, which predominantly encompasses general functionality criteria like ‘ease of use’, ‘navigation’, and ‘gestural design’, our devised scale integrates advanced technology-based wound app functionality measurement criteria.

In this paper, we conducted a comprehensive review of wound apps. To identify apps related to wound assessment and monitoring, a keyword-based search were undertaken in two major app stores (Google Play Store and Apple App Store). The electronic search yielded 170 apps, of which 10 were chosen for evaluation based on our study criteria. Subsequently, the existing app rating scales were adapted to develop one suitable for qualitative and quantitative evaluation of our finalized apps. The research has yielded four key contributions, as outlined below:

- We conducted a thorough evaluation of the existing wound apps available in the two major app stores (i.e., Google Play Store, and Apple App Store).
- We developed a specialized app rating scale tailored for the evaluation of wound assessment apps by extending existing mobile app rating scales.
- We assessed the existing wound apps using our newly developed app rating scale and identified their limitations.
- We discussed future opportunities to harness advanced techniques, particularly those involving artificial intelligence, to enhance the functionality and effectiveness of wound apps.

The remainder of the paper is organized as follows. Section "Methodology" outlines the app search and selection procedure, as well as the devised wound assessment app rating scale. The results of the app evaluations are presented in Section "The Results". Section "Findings and Discussion" discusses the limitations of this study and our findings, including those of the reviewed apps, and future opportunities. Finally, Section "Conclusion and Future Work" offers concluding remarks and suggestions for future research.

## Methodology

### App Search Procedure

We conducted an electronic search to identify relevant apps from two major app stores: Google Play Store and Apple App Store. We employed a keyword-based search process, following similar approaches used in previous studies [33, 34]. The keywords used for searching were: “ulcer” and “wound”. Both app stores were searched using the same keywords to minimize variance and maintain uniformity. This process was carried out independently by three investigators. Each investigator utilized several smartphones to conduct the search and maintained separate lists, which were later merged to form the final app list. Any discrepancies between the lists were resolved through discussion among the investigators.

### App Selection Process

**Fig. 1:**
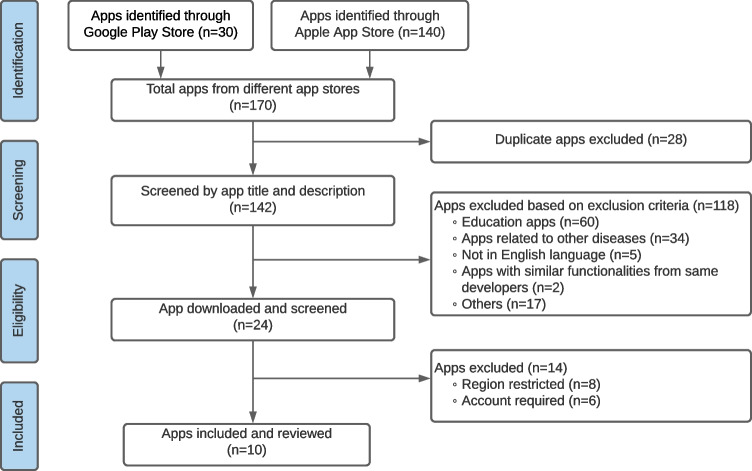
Flow diagram of study methods

We adhered to the Preferred Reporting Items for Systematic Reviews and Meta-Analysis (PRISMA) guidelines [35] for transparency and clarity. Figure 1 illustrates the app screening, eligibility, and selection process.

A keyword-based search yielded 170 apps from the Google Play and Apple App stores, with 140 and 30 apps identified, respectively. We excluded 28 duplicate apps found under the same developer in both stores after confirming identical functionalities on the Android and iOS platforms.

During the screening stage, 142 unique apps were evaluated based on their descriptions in the app store. We excluded 118 apps for various reasons, including educational content, non-English language, relevance to other diseases, and unrelated functionalities. Inclusion criteria focused on wound monitoring, such as tracking healing progress, and assessment capabilities, such as contour detection, measurement, and tissue color classification.

For eligibility screening, 24 remaining apps were downloaded, but 14 required a special account and/or approval from the respective authority, hindering the evaluation (see Appendix A). Finally, 10 apps underwent further assessment, with 8 from the Apple App Store and 2 from Google Play Store. Table 1 provides details of these 10 apps.

**Table 1:** List of wound assessment based apps included in quantitative and qualitative analysis

App name	Country of origin	Platform
ImitoMeasure	Switzerland	Android and iOS
ImitoWound	Switzerland	Android and iOS
Wound Capture	USA	iOS
Wound Doc 3	China	iOS
eKare Insight Health	China	Android and iOS
Wound Measurement	Spain	iOS
SeeWound Online	Sweden	iOS
iX Camera	Canada	iOS
WoundDesk - Wound Care	Germany	Android and iOS
Woundly+	India	Android

### Rating Scale for Wound Apps

We developed a rating scale for evaluating wound apps by building upon and extending existing rating scales such as the Mobile Application Rating Scale (MARS) [32], uMARS [36], a mobile app rating tool for foot measurement (FootMARS) [34], FinMARS [37], and others [38–41]. Although existing rating scales cater to specific domains, there is no established research to evaluate wound apps. Drawing inspiration from these scales, we customized them to suit the requirements of wound assessment apps.

Our rating scale, adapted from FootMARS [34], incorporates changes tailored for wound evaluation apps based on the insights gained from studying their functionality. It encompasses categories such as app classification, aesthetics, general features, performance and efficiency, usability, wound assessment-specific functionality, transparency, and subjective qualities. These domains are crucial for a comprehensive review of wound assessment-based apps.

The app classification category served to collect descriptive data about the app, but was not included in the quantitative analysis. The aesthetics, general features, performance and efficiency, usability, application-specific functionality, transparency, and subjective quality were all incorporated into the app quality evaluation.

We devised a questionnaire comprising 53 questions covering all app quality domains. Irrelevant questions from prior rating scales, such as those concerning social sharing features or user notifications, were excluded. Additionally, the subsection on perceived impact on the user and specific functionality were either modified or omitted to align with the needs of wound assessment-based apps. A five-point Likert scale was employed for 37 questions, with five indicating the highest level of agreement and one representing the lowest level of dissent. Some questions were on a binary scale, but for consistency, these were converted to a Likert scale. An option for “Not Applicable” was provided where certain queries were deemed inappropriate for all apps. To calculate the overall score of this rating scale, the mean of individual app quality domains was computed.

For apps designed to assess wounds, specific functionalities are essential. Accurate wound measurement is critical for analyzing wound severity and estimating healing time. Clinical criteria dictate that the first step in assessing any wound is wound segmentation, separating the wound from surrounding skin and background [42]. Wound measurement aims to track healing progress by measuring changes in length, width, area, circumference, depth, or volume [43, 44]. Subcutaneous tissues become apparent as the wound spreads [44]. Proper diagnosis and wound therapy prescription rely on accurately classifying wound tissue [42]. Temperature measurement is crucial in wound assessment [45], as the healing process may slow if the wound bed temperature drops below core body temperature [46]. Conversely, a rise in temperature indicates wound infection [47]. Thus, in the app-specific functionality domain of our app rating scale, we have considered following factors: i) contour detection of wounds, ii) flexibility in adjusting contour, iii) length and width measurement, iv) area measurement, v) circumference measurement, vi) depth measurement, vii) volume measurement, viii) temperature measurement, ix) tissue color classification, x) visualization of healing, and xi) requirement of servers.

The raters utilized a Likert scale to rate all specific functionality-related evaluations in the app rating scale. The Likert scale, commonly used in survey research, provides a means for respondents to express their level of agreement or disagreement with a given proposition. Our Likert scale comprised five points, with a rating of 5 indicating the best version of a feature, rating 4 signifying a moderately decent version, and rating 3 indicating a point of equilibrium, neither good nor bad. Ratings of 2 and 1 denote progressively worse versions of a feature. Table 2 outlines the app functionality measurement criteria and their corresponding ratings.

**Table 2:** App functionality measurement criteria and their ratings

Measurement criteria	Rating 5	Rating 4	Rating 3	Rating 2	Rating 1
Contour detection	Fully automatic	Partially automatic	Manually with less user input	Complete manually	Does not support
Flexibility of adjusting contour	Excellent	Good	Fair	Poor	Does not support
Length and width measurement	Fully automatic	Automatic but need marker/user input	Manually without marker	Manually but need marker	Does not support
Area measurement	Fully automatic	Automatic but need marker/user input	Manually without marker	Manually but need marker	Does not allow
Circumference measurement	Fully automatic	Automatic but need marker/user input	Manually without marker	Manually but need marker	Does not allow
Depth measurement	Based on 2D (single) image	Based on 2D (multiple) image	Using in-build depth camera	Yes, but need external depth camera	Does not allow
Volume measurement	Based on 2D (single) image	Based on 2D (multiple) image	Using in-build depth camera	Yes, but need external depth camera	Does not allow
Temperature measurement	Without external device	Need external device	-	-	Does not allow
Tissue color classification	With excellent visualization/graph	No visualization (just color value/percentage)	-	-	Does not allow
Visualization of healing	With wound images and graphs	With wound images	With only graphs	-	Does not allow
Requirement of servers	Not at all - completely offline	-	partially - some cases	-	Does not allow

For instance, the ability to detect the contour of a wound was rated on a scale from 5 to 1, reflecting the varying levels of capability across different apps. Fully automatic contour detection received a rating of 5, supported by numerous studies [48–50]. Partially automatic contour detection received a rating of 4, while apps allowing manual contour detection with minimal user input were rated 3. Apps relying entirely on manual contour detection were rated 2, and those lacking contour detection functionality received a rating of 1. Similarly, the feasibility of adjusting the contour of a wound was rated on a scale from 5 to 1, with a rating of 5 indicating excellent feasibility. Ratings of 4 and 3 were assigned to apps with “Good” and “Fair” feasibility, respectively. Apps with poor feasibility received a rating of 2, while those lacking this feature were rated 1.

In any wound assessment-based app, measuring dimensions such as length, width, area, and circumference is crucial. Technologies like digital camera photography [51], Android system photography [52], and others have enabled contactless wound measurement methodologies via photo capture for smart wound size measurement. Additionally, various studies have automated the measurement of wound dimensions without physical contact [53–56]. Apps capable of automatically measuring these metrics receive a rating of 5. Rating 4 denotes apps capable of automatic measurements but requiring a marker or user input. Apps able to perform manual measurements without a marker receive a rating of 3, while those requiring a marker are rated 2. Apps lacking these measurement capabilities receive a rating of 1.

To accurately assess wounds, measuring depth and volume is crucial. Previous studies [57–59] have explored methods for measuring these parameters using images. Apps capable of automatically measuring depth and volume from a single wound image received a rating of 5. Those able to measure from multiple photos received a rating of 4, recognizing the inconvenience of capturing multiple images. Apps using an in-built depth camera were rated 3, while those relying on an external depth camera received a rating of 2. Apps lacking the capability to measure depth or volume were rated 1.

Skin temperature plays a crucial role in wound healing by regulating cell and tissue metabolism [58]. Apps capable of measuring wound temperature without an external device were rated 5, while those requiring an external device received a rating of 4 due to user inconvenience. Apps lacking this feature were rated 1.

Tissue classification is vital for evaluating wound healing. Previous studies have investigated methods for classifying wound tissue [58–61]. Apps that excel in representing wound tissue classification, with graphical representation, received a rating of 5. Rating 4 indicates apps capable of displaying only percentages or values of different tissue colors. Apps lacking this feature received a rating of 1.

Monitoring wound healing poses challenges for clinicians and nurses who must check wounds regularly [62]. Visualizing healing progress aids in understanding improvements. Apps capable of visualizing progress with both ulcer images and graphs were rated 5, while those only displaying ulcer images received a rating of 4. Apps using only graphs received a rating of 3. Apps lacking this feature received a rating of 1.

The requirement for server usage is also considered. Apps capable of functioning entirely offline were rated 5, while those functioning partially offline received a rating of 3. Apps unable to save data, whether in the cloud or on the device, were rated 1.

### Bias Assessment

Each app was assessed using our devised app rating scale/tool by three individual raters (i.e., investigators). To evaluate the results for each app, we collated the average scores for each sub-scale by calculating the scores from each question in each sub-scale. The scores used in the calculation were derived from all raters’ assessments and then discussed as a group to reach a consensus.

The rating tool and the rating quality of raters were examined through internal consistency and inter- and intra-rater reliability, respectively. Internal consistency of the sub-scales of our modified rating scale was calculated to measure the consistency of the scale items. Internal consistency measures the degree of inter-relationships or homogeneity among the items on a test (in our case, the questions/items used in a sub-scale/assessment criteria), such that the items are consistent with one another and measure the same construct [63]. We used Cronbach’s alpha, the most popular means of calculating internal consistency [64]. Cronbach’s alpha ($\alpha$) reliability coefficient indicates internal consistency, ranging between 0 and 1, with $0.9 \le \alpha$ as excellent, $0.8 \le \alpha < 0.9$ as good, $0.7 \le \alpha < 0.8$ as acceptable, $0.6 \le \alpha < 0.7$ as questionable, $0.5 \le \alpha < 0.6$ as poor, and $\alpha < 0.5$ as unacceptable [65]. The closer the value is to 1, the higher the internal consistency. We randomly selected two apps, **ImitoWound** and **eKare Insight Health**, for determining internal consistency. Our revised rating scale’s overall internal consistency was high, with an alpha value of 0.73, which is regarded as acceptable [65].

Inter-rater reliability quantifies the level of agreement between two or more raters who independently rate an item (in this case, an app) based on a set of criteria [66]. We used the intra-class correlation (ICC) method to assess inter-rater reliability. ICC is one of the most widely used statistics for evaluating inter-rater reliability when a study includes two or more raters [67]. In our study, all apps were rated by the same three raters. Thus, we used the ICC two-way mixed model, as it is recommended when the raters are fixed and each of the apps is rated by all raters [68]. Depending on the 95% confidence interval of the ICC estimation, values smaller than 0.5, within 0.5 and 0.75, within 0.75 and 0.9, and higher than 0.90 suggest poor, moderate, good, and excellent reliability, respectively [68]. The ICC score of our final ten apps was calculated as 0.98 (95% CI ranging from 0.98 to 0.99), indicating an excellent level of inter-rater reliability.

Intra-rater reliability measures how consistent an individual is at measuring a set of criteria over time. This reliability estimation involves the same rater performing the same evaluation on more than one occasion. To measure the intra-rater reliability of the three raters, we randomly selected two apps from our included list of ten apps. These two apps were **eKare Insight Health** and **Woundly+**. The three raters reviewed these two apps twice over two months. All three raters demonstrated a significant level of intra-rater reliability between their two ratings, with two-way mixed ICC values of 0.95 (95% CI 0.92–0.95), 0.94 (95% CI 0.90–0.97), and 0.93 (95% CI 0.93–0.97), respectively.

## The Results

### Target Users and Technology Integration

In our assessment of wound apps, we evaluated three key factors: target user, technology, and external dependencies. For target users, we distinguished whether the apps were designed for doctors or patients.

Under the technology category, we analyzed four sub-categories: image processing (IP), 3D or video processing (VP), augmented reality (AR), and machine learning (ML). We recognize the significance of wound images for accurate diagnosis, with 3D shapes offering benefits such as improved measurement accuracy and avoidance of cumbersome procedures like molding or serum injection [69]. Advanced visualization techniques, including augmented, mixed, and virtual reality, further enhance medical experiences [70]. Moreover, machine learning technologies have been extensively employed in wound assessment apps [49, 71].

Additionally, we noted that some wound assessment apps require tools such as sensors and markers for precise measurement. While these accessories can enhance measurement accuracy, they may also pose usability challenges for users.

Table 3 summarizes our assessment results for the 10 selected apps. All the reviewed apps were designed specifically for use by doctors or wound assessment experts, with none intended for patient use. We found that all apps utilized 2D image-based technology for wound measurement, while some incorporated video or 3D models. Apps such as **Wound Capture** and **Wound Measurement** utilized augmented reality, while others like **Wound Capture**, **SeeWound Online**, and **Woundly+** employed machine learning algorithms. Furthermore, we noted that the majority of current apps require external sensors or markers for wound measurement, which may adversely affect their usability.

**Table 3:** Target user and technology integration of wound apps

App name	Target user		Technology				Additional accessories	
	Practitioner	Patient	IP	VP	AR	ML	Sensor	Marker
ImitoMeasure	✓	✗	✓	✗	✗	✗	✗	✓
ImitoWound	✓	✗	✓	✗	✗	✗	✗	✓
Wound Capture	✓	✗	✓	✗	✓	✓	✓	✗
Wound Doc 3	✓	✗	✓	✗	✗	✗	✗	✓
eKare Insight Health	✓	✗	✓	✓	✗	✗	✓	✗
Wound Measurement	✓	✗	✓	✗	✓	✗	✗	✗
SeeWound Online	✓	✗	✓	✗	✗	✓	✗	✓
iX Camera	✓	✗	✓	✓	✗	✗	✗	✓
+WoundDesk	✓	✗	✓	✗	✗	✗	✗	✓
Woundly+	✓	✗	✓	✗	✗	✓	✗	✓

*IP* Image processing, *VP* Video processing, *AR* Augmented Reality, *ML* Machine learning

### Assessment of Functionality

**Fig. 2:**
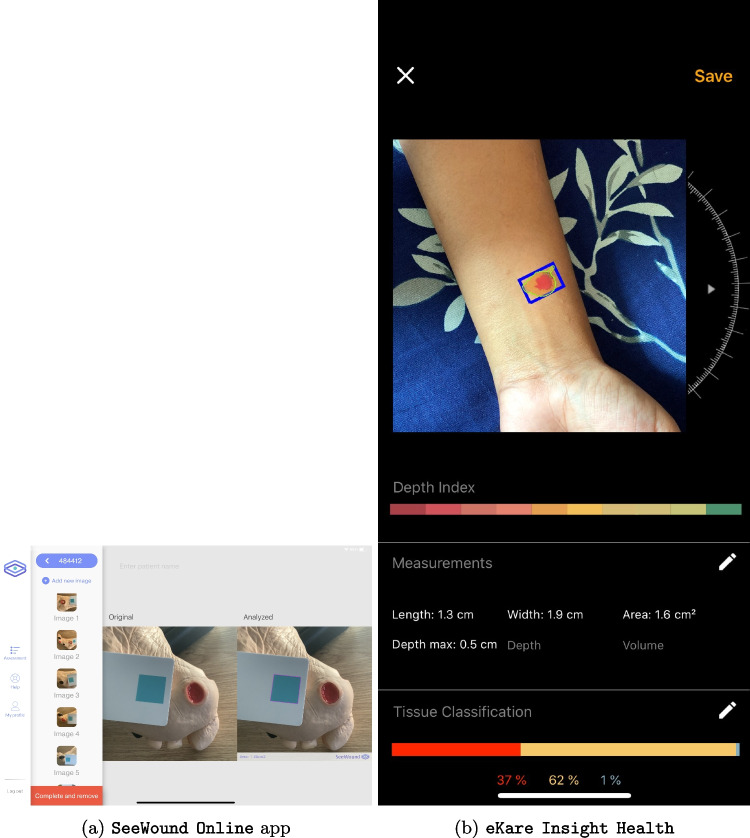
Apps screenshot (**a**) SeeWound Online app demonstrating contour detection (**b**) eKare Insight Health app demonstrating depth measurement and tissue classification

Table 4 summarizes the evaluation of app functionality based on the criteria outlined in Table 2. Among the 10 apps assessed, **SeeWound Online** is the only app that automatically detects wound contours effectively, as depicted in Fig. 2a. In contrast, **Wound Capture** and **Wound Measurement** lack contour detection capabilities entirely, while the other six apps only offer partial support for this feature. In terms of automatically measuring wound length and width, only **imitoMeasure** and **Wound Measure** excel, while **SeeWound Online** and **Woundly+** lack this functionality. Similarly, **Wound Capture** and **Wound Measurement** do not offer area measurement capabilities, unlike **SeeWound Online** and **imitoMeasure**.

**Table 4:** Result of App functionality Assessment

Measurement criteria	Rating 5	Rating 4	Rating 3	Rating 2	Rating 1
Contour detection	1 (10%)	6 (60%)	0	1 (10%)	2 (20%)
Flexibility of adjusting contour	4 (40%)	1 (10%)	1(10%)	0	4 (40%)
Length and width measurement	2 (20%)	6 (60%)	0	0	2 (20%)
Area measurement	2 (20%)	6 (60%)	0	0	2 (20%)
Circumference measurement	1 (10%)	3 (30%)	0	0	6 (60%)
Depth measurement	0	0	1 (10%)	1 (10%)	8 (80%)
Volume measurement	0	0	0	1 (10%)	9 (90%)
Temperature measurement	0	1 (10%)	–	–	9 (90%)
Tissue color classification	2 (20%)	0	–	–	8 (80%)
Visualization of healing	1 (10%)	1 (10%)	3 (30%)	–	5 (50%)
Requirement of servers	8 (80%)	–	0	–	2 (20%)

Circumference measurement, crucial for understanding the healing process, is present in only 40% of the evaluated apps, with **imitoMeasure** being the standout performer. Adjusting contour flexibility is a common feature, present in 60% of the apps, with **Wound Doc 3** and **imitoMeasure** among the top performers.

Depth measurement and tissue color classification features are scarce, present in only 20% of the apps, with **eKare Insight Health** (see Fig. 2b) and **Wound Measurement** being the exceptions. Volume and temperature measurement functionalities are largely absent, with only **eKare Insight Health** offering volume measurement and **Wound Capture** providing temperature measurement.

Healing progress visualization is available in 50% of the apps, with **Wound Capture** and **ImitoWound** featuring this functionality. Overall, **eKare Insight Health** offers the most comprehensive range of functionalities, meeting 10 out of 11 criteria, followed by **Wound Doc 3**, **imitoWound**, and **imitoMeasure**. In contrast, **Wound Capture** and **Woundly+** lag behind, meeting only 4 criteria out of 11.

The majority of the apps fail to meet several specific functionality requirements, particularly in measuring depth and volume and showing wound healing progress. Tissue color classification is also lacking in 80% of the apps.

While we have reviewed all 10 apps, including screenshots of each app would not add significant value to our discussion. Instead, we focus on illustrating particular functionalities that exemplify the technological advancements in wound care applications. This targeted approach allows us to provide a deeper insight into the unique contributions of these apps. By concentrating on these specific examples, we aim to highlight the diversity and innovation within the field without suggesting bias towards these two applications. Our selection is based solely on the distinctiveness of their features, which are pertinent to the context of our study.

### Evaluation Scores

In Table 5, we present the mean and standard deviation of each app’s sub-scales scores, as well as their overall score. The app with the lowest score, **Wound Measurement**, achieved an overall mean score of 2.39, indicating poor quality and ineffectiveness in wound measurement. Conversely, **ImitoWound** received the highest mean score of 4.24, demonstrating its superiority across all rating sub-scales.

Performance and efficiency, usability, and transparency domains showed better rankings compared to others, with mean scores of 4.43, 3.55, and 3.73, respectively. In contrast, application-specific functionality and subjective quality domains received the lowest mean ratings of 2.55 and 3.03, respectively. Notably, app performance and efficiency received the highest mean score of 4.43.

**Table 5:** Evaluation scores for wound apps

App name	Aesthetics	Performance	Usability	Functionality	Transparency	Subjective	Mean (Std dev)
eKare Insight Health	3.25	4.25	3.50	3.45	4.25	3.67	3.73 (0.43)
Wound Capture	3.50	4.25	3.75	2.27	4.50	2.67	3.49 (0.87)
Wound Doc 3	5.00	4.37	3.50	3.36	4.75	4.33	4.22 (0.66)
Woundly+	2.25	3.75	2.50	2.09	2.50	1.33	2.40 (0.79)
imitoMeasure	4.00	4.75	5.00	3.09	4.25	4.00	4.18 (0.67)
+WoundDesk	4.25	4.75	4.25	2.36	4.25	4.00	3.98 (0.83)
ImitoWound	5.00	4.87	4.50	3.09	4.00	4.00	4.24 (0.70)
iX Camera	3.00	4.62	3.50	2.18	4.00	3.00	3.38 (0.86)
Wound Measurement	1.00	4.12	2.75	1.90	2.25	2.33	2.39 (1.03)
SeeWound Online	2.50	4.50	2.25	1.72	2.50	1.00	2.41 (1.17)

**Fig. 3:**
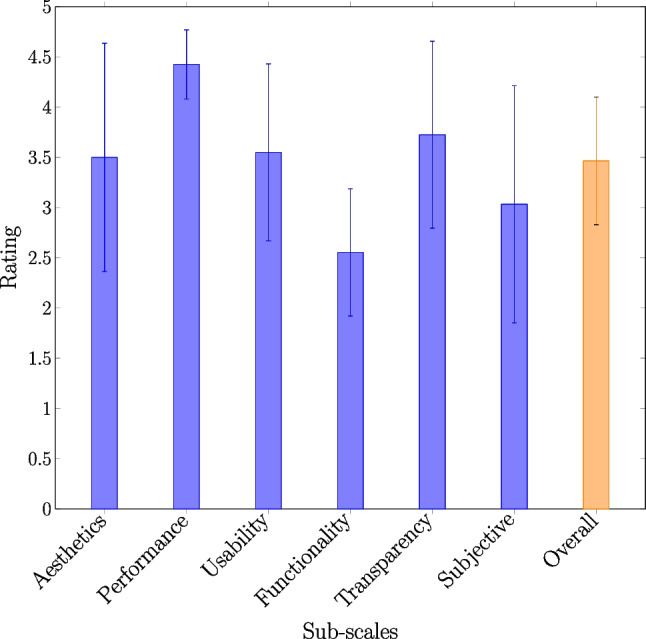
Sub-scale specific ratings and overall rating

These scores were utilized to calculate both domain-specific and overall app ratings, as depicted in Fig. 3. The average app rating across all apps was 3.46 out of 5, indicating a moderate level of performance and quality.

## Findings and Discussion

### Limitations of Reviewed Apps

Despite the abundance of wound assessment apps, many lack critical features deemed essential in our study, such as the ability to measure length, width, depth, volume, temperature, and tissue classification. Only one app, **See Wound Online**, was capable of automatically detecting the contour of a wound, while most apps required markers for this purpose. Obtaining and using these markers proved cumbersome for users, as different apps often required different markers. Additionally, the absence of markers often restricted further wound measurements in some apps.

Depth measurement, a vital aspect of wound assessment, was lacking in 80% of the reviewed apps, rendering volume measurement impossible in their absence. Only **eKare Insight Health** could measure wound volume, albeit requiring an external depth camera. Similarly, temperature measurement, crucial for wound infection diagnosis and healing rate assessment, was only possible in **Wound Capture**, albeit requiring an external device.

Tissue classification, vital for determining wound severity, was absent in 80% of the reviewed apps, and only half of them displayed wound healing progress. None of the evaluated apps met all critical parameters for wound assessment.

Despite the rapid advancements in mobile technology, most reviewed apps did not leverage advanced technologies. Video or 3D image processing, powerful for automatic wound assessment, was utilized in only 20% of the apps, with **eKare Insight Health** and **iX Camera** being exceptions. Augmented Reality, beneficial for dermatological measurements, was present in only two apps, namely **Wound Capture** and **Wound Measurement**. Additionally, only three apps utilized machine learning, a technology with significant potential for improving wound assessment accuracy.

Moreover, the majority of apps targeted healthcare professionals, utilizing medical terminology that may be challenging for the general public to comprehend. Thus, there is a need for the development of patient-focused apps with simplified language to enhance accessibility and understanding.

### Opportunities

In this study, we identified significant functional gaps in wound assessment-based apps, highlighting the need for functional enhancements leveraging advancements in artificial intelligence and related research. Despite extensive research on wound contour detection, most existing apps lack automated functionality in this regard [48–50]. Similarly, although numerous studies have explored automatic measurement of wound dimensions such as length, width, area, and circumference, few apps incorporate these features [54–56]. Additionally, research on automating wound depth and volume measurement, skin temperature assessment, and tissue classification presents opportunities for future app development [57–59].

Several studies have employed machine learning and deep learning algorithms for wound area detection, segmentation, and tissue classification [72–79]. Similarly, 3D modeling techniques have been utilized to extract wound features such as width, length, area, depth, and volume [58, 62, 80], and to monitor healing [81]. Augmented Reality (AR), although underutilized in current apps, shows promise for enhancing user experiences in wound assessment and health science education [82–84]. Furthermore, various sensor-based approaches [85], such as LiDAR camera [86], have been explored for wound assessment, yet most current apps do not leverage sensor technologies [87–89]. Given the potential of these technologies and the advancements in smartphone capabilities, future wound assessment apps should integrate these features.

Visual appeal and usability are crucial factors for app success. However, our findings indicate that many reviewed apps scored poorly in these domains [90]. Enhancing the visual appeal and usability of wound assessment apps is imperative for improving user satisfaction and engagement. Aesthetic improvements can enhance user experience and attract a broader audience, while improved usability ensures efficient task completion and user satisfaction [91]. Therefore, future app development efforts should prioritize visual design and usability enhancements to meet user expectations and preferences.

One notable limitation is the lack of apps designed specifically for patients. While the assessed apps primarily target healthcare professionals, there is a notable gap in apps tailored for patient use. Patient-focused apps are crucial for empowering individuals to actively participate in their wound care management, facilitating better communication with healthcare providers, remote monitoring, and enhancing overall patient engagement and satisfaction.

### Limitations of This Study

Several limitations must be acknowledged in this study. Firstly, our analysis was restricted to wound assessment applications available in English, potentially excluding apps in other languages that may offer valuable features. Secondly, since our app search and evaluation, some of the apps may have been updated with improved features, been removed from the app stores, or new apps may have been added. Additionally, apps with region-based access restrictions were not included, limiting the comprehensiveness of our assessment. Some apps required user account approval, which we attempted to obtain but encountered challenges with non-responsive authorities, leading to the exclusion of potentially relevant apps. The inability to assess these apps may have impacted the breadth of our findings.

Furthermore, the use of different mobile devices with varying operating systems by the raters resulted in differences in app performance and ratings for certain criteria. This variability may have influenced the consistency of our evaluations. Additionally, while our study focused on the presence of functionality in wound assessment apps, we did not assess the accuracy or reliability of these functions. Therefore, the effectiveness of the identified features was not evaluated, which may limit the applicability of our findings in real-world clinical settings. These limitations should be considered when interpreting the results of our study.

## Conclusion and Future Work

In this study, we conducted a systematic review of mobile apps available on major app stores, resulting in the identification of 170 relevant apps. Subsequently, we rigorously evaluated and rated 10 selected apps using our modified app rating scale, specifically designed for assessing wound assessment-based apps. Despite the wide array of available apps, our findings revealed significant shortcomings in their functionality and design.

Our research uncovered that the majority of existing mobile apps do not fully meet the necessary criteria for accurately assessing human body wounds. While some apps offered certain expected features, none of them encompassed all the required functionalities. This highlights a critical gap in the current landscape of wound assessment apps and underscores the need for further development and improvement in this domain.

Moving forward, future research should focus on addressing the identified design flaws and enhancing the functionality of wound assessment apps. This could involve leveraging advanced technologies such as image/video processing, machine learning, and augmented reality to improve the accuracy and efficiency of wound assessment. Moreover, there is a pressing need for rigorous validation studies to assess the reliability and effectiveness of the identified factors (in particular, wound assessment-specific functionality) in real-world clinical settings. By collaborating with healthcare professionals and researchers, developers can ensure that these apps meet the standards required for clinical use and contribute to improved patient outcomes.

In conclusion, while the current state of wound assessment apps may fall short of expectations, ongoing advancements in technology and research present promising opportunities for the development of more comprehensive and effective solutions in the future.

## Data Availability

No datasets were generated or analysed during the current study.

## Appendix A

### List of Wound Apps that Required Approved Membership to be able to use

**Table 6:** List of wound apps that were not accessible

App name	
PointClickCare Skin and Wound	
Wound Tracker Professional	
Swift Skin and Wound	
InteliWound	
Swift Skin and Wound Training	
Medline	
WoundVision	
Healogics Photo	
Collab Care	
Tissue Analytics	
Parable	
Rita your wound care expert	
My Wound Doctor	
SeeWound	
